# The association of cancer‐related fatigue with all‐cause mortality of colorectal and endometrial cancer survivors: Results from the population‐based PROFILES registry

**DOI:** 10.1002/cam4.2166

**Published:** 2019-04-22

**Authors:** Salome Adam, Lonneke V. van de Poll‐Franse, Floortje Mols, Nicole P. M. Ezendam, Ignace H. J. T. de Hingh, Volker Arndt, Melissa S. Y. Thong

**Affiliations:** ^1^ Netherlands Comprehensive Cancer Organisation Utrecht the Netherlands; ^2^ Division of Chronic Disease Epidemiology, Epidemiology, Biostatistics and Prevention Institute University of Zurich Switzerland; ^3^ Department of Medical and Clinical Psychology, CoRPS—Center of Research on Psychology in Somatic Diseases Tilburg University Tilburg the Netherlands; ^4^ Division of Psychosocial Research and Epidemiology The Netherlands Cancer Institute Amsterdam the Netherlands; ^5^ Catharina Cancer Institute, Catharina Hospital Eindhoven the Netherlands; ^6^ Unit of Cancer Survivorship, Division of Clinical Epidemiology and Aging Research German Cancer Research Center (DKFZ) Heidelberg Germany; ^7^ Department of Medical Psychology Amsterdam Public Health Research Institute, Location AMC, Amsterdam UMC Amsterdam the Netherlands

**Keywords:** all‐cause mortality, cancer‐related fatigue, colorectal cancer, endometrial cancer

## Abstract

**Purpose:**

Cancer‐related fatigue (CRF) is one of the most prevalent symptoms experienced by cancer survivors. However, researchers are only beginning to elucidate the risk factors, underlying mechanism(s), and its association with other outcomes. Research on the association between CRF and mortality is limited.

**Methods:**

The study sample comprised 2059 short‐term (<5 years postdiagnosis) cancer survivors from four PROFILES registry studies. Survivors diagnosed with stage I‐III colorectal cancer (CRC) or stage I‐III endometrial cancer (EC), with no evidence of disease, were identified and followed‐up by the Netherlands Cancer Registry. Fatigue was assessed with the Fatigue Assessment Scale. Cox proportional hazards models adjusted for demographic, clinical, and lifestyle characteristics were performed to assess the association of CRF with all‐cause mortality. Date of censoring was February 1, 2017.

**Results:**

Prevalence of CRF varied between 35.8% (male CRC) and 43.6% (female CRC). After a median follow‐up period of 9.0 years, a total of 408 survivors (20%) had died. CRF was associated with increased all‐cause mortality in male CRC survivors (HRadj = 1.75, 95% CI [1.31‐2.33]). This association remained statistically significant after excluding survivors experiencing anhedonia. For female CRC (HRadj = 1.32, 95% CI [0.90‐1.97]) and EC (HRadj = 1.27, 95% CI [0.84‐1.90]) survivors, there was no significant association with all‐cause mortality for the fatigued group in multivariable analyses.

**Conclusion:**

Our study found that CRF is significantly associated with all‐cause mortality in male CRC survivors, irrespective of potential confounders. This result suggests that clinicians should increase their attention towards the recognition and treatment of CRF.

## 1 INTRODUCTION

Cancer‐related fatigue (CRF) is a “distressing persistent subjective sense of physical, emotional, and/or cognitive tiredness or exhaustion related to cancer or cancer treatment that is not proportional to recent activity and that interferes with usual functioning”.^1^ Moreover, CRF is one of the most prevalent symptoms experienced by cancer survivors.^2^

However, researchers are only at the beginning to elucidate the risk factors and underlying mechanism(s) of CRF, and other outcomes associated with CRF.^3^ Research shows that in breast and testicular cancer survivors, CRF has been positively correlated with psychological distress, lifestyle, and clinical factors.^4^, ^5^ Additionally, in breast cancer survivors, CRF can have a significant negative impact on survivors’ quality of life.^6^ There is also some evidence suggesting that CRF is a significant prognostic factor for survival for prostate and breast cancer.^7^, ^8^ But for colorectal cancer (CRC), studies reveal contradictory results. Two studies could not find an association between CRF with survival,^9^, ^10^ whereas one showed an association.^11^ For endometrial cancer (EC), a univariate association between CRF and survival disappeared after adjustment for confounders.^12^ These results show that currently research into the association between CRF and mortality is limited and the results are contradictory.

As such, a better understanding of the association between CRF and mortality is important as many cancer survivors are living longer thanks to earlier detection and better treatment.^13^ These trends are also observed for CRC and EC. CRC is the third most common cancer and EC is the most common gynecological cancer.^14^ A significant proportion of this growing number of survivors will remain fatigued after active treatment has ended; studies show that between 35% and 40% of short‐term (<5 years) CRC and EC survivors and 35% of long‐term CRC survivors are fatigued.^15^, ^16^, ^17^

Therefore, our study aimed to explore whether CRF is associated with all‐cause mortality in a large population‐based sample of short‐term (<5 years since diagnosis) EC and CRC survivors (with no evidence of disease). As CRF prevalence and mortality rates vary by gender, we assessed the association of CRF with all‐cause mortality stratified by gender.^18^, ^19^ Additionally, we investigated whether psychological or clinical factors influence a potential association.

## 2 METHODS

### 2.1 Setting and participants

This study pooled data from four large population‐based patient‐reported outcome surveys on CRC and EC survivors conducted between January 2008 and December 2012. These surveys are part of the PROFILES (‘Patient Reported Outcomes Following Initial treatment and Long‐term Evaluation of Survivorship’) registry.^20^ Patient Reported Outcome (PRO) data are collected in PROFILES within a sampling frame of the Netherlands Cancer Registry (NCR) and can be linked with clinical data of all individuals newly diagnosed with cancer in the Netherlands.

Eligible participants for this analysis were short‐term (<5 years postdiagnosis) stage I‐III CRC or stage I‐III EC survivors, diagnosed between 2003 and 2012. Exclusion criteria included cognitive impairment, death prior to start of study (according to the ECR, the Central Bureau for Genealogy and hospital records) or unverifiable addresses. Additionally, all CRC survivors with a confirmed diagnosis of metachronous metastasis or local recurrence were excluded.^21^

### 2.2 Data collection

A detailed description of the data‐collection has been published previously.^20^ Briefly, in each study sample, eligible cancer survivors were informed about the study via a letter by their (previous) attending specialist. Invited study participants were given the option of completing either an online or paper questionnaire. Nonrespondents were sent a reminder and questionnaire after 2 months. Data from the PROFILES registry are freely available for noncommercial scientific research, subject to study question, privacy and confidentiality restrictions, and registration (www.profilesregistry.nl).

Separate ethical approval for the four studies was obtained from local certified Medical Ethics Committees in the Netherland, approval numbers: MMC Veldhoven 0733, 0822, NL33429.008.10. Written informed consent was obtained from all participants. All procedures involving human participants were in accordance with the Helsinki Declaration of 1975, as revised in 1983.

### 2.3 Study measurements

#### 2.3.1 Fatigue assessment scale (FAS)

This 10‐item Dutch validated questionnaire^22^ assesses how patients usually feel about their fatigue. It has good psychometric properties and has been used previously with cancer patients.^23^ Responses were arranged on a five‐point scale (1: never to 5: always). To indicate the level of fatigue, the score can be categorized either dichotomously: not fatigued (FAS‐score: 10‐21) and fatigued (FAS‐score: 22‐50) or in tertiles: not fatigued (FAS‐score: 10‐21), fatigued (22‐34), and very fatigued (≥35).^22^, ^23^

#### 2.3.2 Anhedonia

To reduce the possible overlap of physical symptoms of depression with fatigue, we used items from the Hospital Anxiety and Depression Scale (HADS) that assessed lack of positive affect (ie, anhedonia).^24^ This subscale consists of four items*: I look forward with enjoyment to things, I feel cheerful, I can laugh and see the funny side of things,* and *I still enjoy the things I used to enjoy* (range 0‐12, mean + SD 2.2 ± 2.3). We defined anhedonia using a cut‐off score of ≥ 6 (ie, one SD above the mean) from the total score of the four items.^25^

#### 2.3.3 Demographic, lifestyle, and clinical data

The NCR provided data on demographic and clinical information including date of birth, date of diagnosis, cancer stage, primary treatment (radio‐ and chemotherapy), and vital status.

For the CRC sample, information on metachronous metastasis (defined as distant metastasis of primary CRC in other organs, excluding regional lymph nodes) and local recurrence were derived from an additional data collection, performed between 2010 and 2011 for a subgroup of CRC survivors with time of diagnosis similar to that of the current study sample. Further details are explained elsewhere.^21^

Self‐reported demographic data included marital status, education, weight, and height. BMI was calculated with self‐reported height and weight. Information on self‐reported lifestyle factors included smoking and alcohol usage. Comorbidity at time of survey was assessed with the adapted Self‐administered Comorbidity Questionnaire.^26^

### 2.4 Statistical analyses

We compared clinical (stage, primary treatment, time since diagnosis, number of comorbidities at survey, and BMI) and sociodemographic (age, sex, education, smoking, alcohol drinking, and marital status) characteristics by gender of CRC survivors and compared female CRC with EC survivors using parametric tests (eg, ANOVA) or nonparametric equivalents (eg, Kruskal‐Wallis test).

Cox proportional hazard models, with two‐sided 95% confidence intervals (CIs) for the hazard ratios (HRs), were performed to assess the association of CRF with all‐cause mortality. We specified the survival duration as time from the invitation to study until either death or censoring date (February 1, 2017). The models were adjusted for age at invitation, tumour stage, primary treatment, years since diagnosis, number of comorbidities at survey, education, and smoking. Additionally, we adjusted for potential survivorship bias by adding a variable with the left‐truncation time (time between diagnosis and study invitation) and we set time of study invitation as entry time. The proportional hazards requirement, assuming that the HR was constant over time, was visually checked using log‐log plots, and violation of the requirement was assumed when the lines were not parallel.

Additionally, a sensitivity analyze was performed using the same Cox proportional hazards models, excluding cancer survivors who reported symptoms of anhedonia.

To address the possible bias due to missing values, multiple imputation (Multiple Imputation Chained Equations with 25 imputations^27^, ^28^) was employed. All statistical analyses were conducted using the Stata version 13.1.

## 3 RESULTS

### 3.1 Survivors characteristics

From the four data collections, 2848 eligible survivors received a study invitation and 789 did not answer (response rate = 72.3%). After exclusion of survivors with nonverified address, comparisons between respondents and nonrespondents showed that nonrespondents were significantly older (*P* < 0.001), were more likely to be female (*P* = 0.007) and had more often cancer stage II (*P* = 0.010) (Table S1).

In the final sample 2059 respondents were included, of which 70.9% and 29.1% were CRC and EC survivors, respectively. As of February 1, 2017, 408 (20%) respondents had died. The median follow‐up time between time of survey completion to time of follow‐up was 9.0 years (range 0.4‐13.8 years).

HRs of clinical characteristics of respondents revealed that older cancer survivors had a significantly increased risk of all‐cause mortality (Table 1). Years since diagnosis and cancer stage were predominantly not significantly associated with all‐cause mortality when focusing on HRs stratified by cancer type and gender. CRC survivors treated with radiotherapy reported a reduced risk of all‐cause mortality, however, the HR was only significant in female CRC survivors. Overall, cancer survivors with two or more reported comorbidities had a significant increased risk of all‐cause mortality. Moreover, cancer survivors with a comorbid heart condition had in all subgroups an increased mortality risk, whereas no significant association could be seen for high blood pressure and arthritis. No clear pattern for an association of education level and BMI could be seen, whereas being married/cohabited significantly reduced the risk. Being a former or current smoker significantly increased the all‐cause mortality risk in male CRC survivors, though no association could be seen in female CRC and EC survivors. Finally, alcohol consumption was associated with lower mortality in female and male CRC survivors.

**Table 1:** Baseline characteristics and all‐cause mortality of study participants, stratified by cancer type and gender

	Baseline characteristics				Hazard Ratios (all‐cause mortality)			
	Total	CRC male	CRC female	EC	Total	CRC male	CRC female	EC
	n = 2,059	n = 821	n = 638	n = 600				
	Col%	Col%	Col%	Col%	HR	HR	HR	HR
Age at survey								
≤60	30.5	28.0	27.7	36.8	1.00	1.00	1.00	1.00
61‐70	36.8	37.6	32.5	40.4	2.52*	2.28*	3.12*	2.49*
70+	32.7	34.4	39.8	22.8	6.52*	6.08*	10.70*	5.30*
Mean (SD)	65.2 (9.5)	65.7 (9.3)	65.9 (10.1)	63.7 (8.9)	1.09*	1.09*	1.13*	1.08*
Cancer stage								
I	46.6	31.8	24.8	90.0	1.00	1.00	1.00	1.00
II	31.0	40.0	42.5	6.5	1.31*	1.19	1.03	1.36
III	22.0	28.1	32.5	2.3	1.19	0.99	0.85	9.29*
Unknown	0.4	0.1	0.2	1.2	3.33*	—	—	5.36*
Years since diagnosis								
≤1	15.4	3.9	2.8	44.7	1.00	1.00	1.00	1.00
2‐3	58.1	67.2	69.8	36.4	0.68*	0.78	0.51	0.61
4‐5	26.5	28.9	27.4	18.9	0.56*	0.54	0.47	0.55
Chemotherapy	20.8	26.9	29.8	3.0	0.79	0.75	0.50*	3.50*
Radiotherapy	26.4	28.6	21.3	28.7	1.03	0.73	0.84	1.74*
Number of comorbidities at survey								
0	34.9	40.7	31.5	30.7	1.00	1.00	1.00	1.00
1	33.2	32.2	35.4	32.1	1.05	1.13	1.19	0.89
2	19.9	17.5	21.3	21.8	1.58*	1.82*	1.36	1.69*
≥3	12.0	9.6	11.8	15.4	1.90*	2.09*	2.61*	1.50
Most common comorbid conditions at survey								
Heart condition	15.2	19.4	13.0	11.6	2.06*	1.72*	3.15*	1.65*
High blood pressure	36.1	31.7	33.4	45.2	1.14	1.08	1.14	1.35
Arthritis	27.7	16.8	36.6	33.4	1.07	1.03	1.27	1.23
Education^a^								
Low	17.7	19.9	17.1	15.5	1.00	1.00	1.00	1.00
Medium	60.4	57.0	60.2	65.2	0.75*	0.84	0.63	0.86
High	20.0	21.4	20.2	17.7	1.19	1.02	1.28	1.69
Unknown	1.9	1.7	2.5	1.6	1.34	1.03	2.51*	0.55
Married/Cohabiting (yes)	75.5	84.7	62.2	73.3	0.67*	0.65*	0.58*	0.67*
Unknown	1.5	1.5	2.2	0.8	1.37*	0.83	2.22	1.37*
Body mass index								
<18.5	0.8	0.5	1.6	0.5	1.00	1.00	1.00	1.00
18.5‐24.9	30.6	28.8	35.4	28.1	0.84	0.55	0.70	0.90
25.0‐29.9	43.4	53.8	39.8	32.9	0.72	0.43*	0.80	0.70
≥30	22.2	14.6	18.5	36.5	0.90	0.6	0.63	1.09
Unknown	3.0	2.3	4.7	2.0	1.66	1.03	2.36*	0.86
Smoking^b^								
No	31.6	20.0	46.6	52.5	1.00	1.00	1.00	1.00
No, but used to	54.9	65.5	41.2	34.5	1.36*	2.74*	0.84	0.49
Currently	10.9	11.3	10.3	11.0	1.90*	3.39*	1.68	0.88
Unknown	2.6	3.2	1.9	2.0	0.89	1.88	0.89	1.20
Alcohol consumption^b^								
No	23.3	11.9	37.8	45.5	1.00	1.00	1.00	1.00
No, but used to	5.0	6.7	2.8	2.5	1.03	0.88	0.72	0.93
Currently	53.5	60.9	44.0	38.5	0.55*	0.48*	0.43*	0.70
Unknown	18.2	20.5	15.4	13.5	0.66*	0.61*	0.45*	0.75

Abbreviations: Col., column; CRC, colorectal cancer; EC, endometrial cancer; HR, hazard ratios.

a Education: Low (no or primary school); Medium (lower general secondary education or vocational training); High (preuniversity education, high vocational training, university).

b Lower sample sizes for endometrial cancer as in one substudy this question was not asked.

* *P*‐values indicate significant (*P* < 0.05) association with all‐cause mortality.

**Figure 1:**
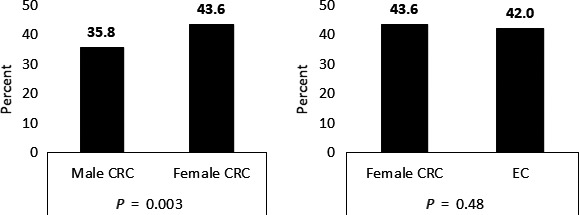
Distribution of CRF (%) in cancer survivors, stratified by cancer type and gender. Fatigue assessment scale (FAS) total score cut‐off: not fatigued (10‐21) and fatigued (22‐50)^22^, ^23^

Prevalence of CRF varied between 35.8% (male CRC survivors) and 43.6% (female CRC survivors). The number of survivors reporting to be fatigued did not differ significantly when female CRC and EC survivors were compared (*P* = 0.48). However, female CRC survivors were more likely to be fatigued than male CRC survivors (*P* = 0.003) (Figure 1). Overall, there were no significant differences between cancer stage, chemo‐ and radiotherapy between not fatigued and fatigued survivors (data not shown).

### 3.2 Association of CRF with all‐cause mortality

Overall, univariate analysis showed that there is a significant association between CRF and all‐cause mortality (HR = 1.56, 95% CI [1.29‐1.90]) (Table S2). The effect also remained statistically significant after adjusting for confounders (HRadj = 1.50, 95% CI [1.19‐1.78]). However, in multivariable analysis it is shown that female cancer survivors have a significant decreased all‐cause mortality risk (HRadj = 0.68 95% CI [0.54‐0.82]). As imputed analyses revealed no substantial differences from those based on nonimputed data, we report subsequent results using the imputed data.

#### 3.2.1 Stratified by cancer type and gender

Using the dichotomous FAS scores, univariate analysis showed that the risk of all‐cause mortality increased significantly in the fatigued group of male CRC survivors (HR = 1.78, 95% CI [1.34‐2.37]) (Table 2). The effect also remained statistically significant after adjusting for confounders (HRadj = 1.75, 95% CI [1.31‐2.33]). In uni‐ and multivariable analyses of female CRC survivors, no significant increased risk of all‐cause mortality could be seen for the fatigued group (HRadj = 1.32, 95% CI [0.90‐1.97]). Also, fatigued EC survivors showed no significant increased risk of all‐cause mortality after adjustment for confounders (HRadj = 1.27, 95% CI [0.84‐1.90]).

**Table 2:** Risk estimates of the association of CRF with all‐cause mortality of cancer survivors, stratified by cancer type and gender, using imputed data

	Total, N	Deaths, N	Person‐years	Univariate		Adjusted^a^	
				HR	95% CI	HR	95% CI
Male colorectal cancer survivors							
Not fatigued	527	100	3,280.3	1.00	—	1.00	—
Fatigued	294	93	1,723.8	1.78	1.34‐2.37	1.75	1.31‐2.33
Female colorectal cancer survivors							
Not fatigued	360	51	2,299.4	1.00	—	1.00	—
Fatigued	278	55	1,719.6	1.45	0.99‐2.13	1.32	0.90‐1.97
Endometrial cancer survivors							
Not fatigued	348	53	2,375.8	1.00	—	1.00	—
Fatigued	252	56	1,655.2	1.51	1.03‐2.19	1.27	0.84‐1.90

FAS total score cut‐offs: not fatigue (10‐21) & fatigue (22‐50).^22^, ^23^

a Analysis was adjusted for age at invitation, cancer stage, primary treatments, years since diagnosis, education, number of comorbidities at invitation and smoking if appropriate.

When stratifying the FAS score into three categories (not fatigued/fatigued/very fatigued), very fatigued male CRC survivors (HRadj = 2.78, 95% CI [1.47‐5.22]) and very fatigued endometrial survivors (HRadj = 2.27, 95% CI [1.06‐4.96]) reported a significant increased all‐cause mortality risk in multivariable analysis (Table 3). These HRs were distinctly higher than the HRs for fatigued cancer survivors in both groups. In female CRC survivors very fatigued survivors (HRadj = 1.27, 95% CI [0.58‐2.80]) reported a lower and not significant HR than fatigued survivor.

**Table 3:** Risk estimates of the association of CRF with all‐cause mortality of cancer survivors, stratified by cancer type and gender, using imputed data

	Total, N	Deaths, N	Person‐years	Univariate		Adjusted^a^	
				HR	95% CI	HR	95% CI
Male colorectal cancer survivors							
Not fatigued	526	100	3,274.3	1.00	—	1.00	—
Fatigued	263	81	1,559.5	1.61	1.28‐2.30	1.64	1.22‐2.22
Very fatigued	31	12	164.3	2.95	1.35‐4.48	2.78	1.47‐5.22
Female colorectal cancer survivors							
Not fatigued	360	51	2,299.4	1.00	—	1.00	—
Fatigued	243	47	1,497.9	1.74	0.96‐2.11	1.50	0.80‐1.85
Very fatigued	35	8	216.1	1.38	0.80‐3.56	1.27	0.58‐2.80
Endometrial cancer survivors							
Not fatigued	348	53	2,375.8	1.00	—	1.00	—
Fatigued	226	47	1,491.8	1.40	0.94‐2.07	1.24	0.82‐1.90
Very fatigued	26	9	163.4	2.55	1.26‐5.17	2.27	1.06‐4.96

FAS total score cut‐offs: not fatigued (10‐21), fatigued (22‐34), very fatigued (≥35).^22^, ^23^

a Analysis was adjusted for age at invitation, cancer stage, primary treatments, years since diagnosis, education, number of comorbidities at invitation, and smoking if appropriate.

### 3.3 Subgroup analysis

Pre‐planned subgroup analysis in 1453 (70.6%) survivors not experiencing anhedonia showed that among male CRC survivors, being fatigued (HRadj = 1.74, 95% CI [1.23‐2.48]) was associated with higher mortality risk (Table 4). Fatigued female survivors of CRC not experiencing anhedonia (HRadj = 1.11, 95% CI [0.70‐1.77]) or EC (HRadj = 1.38, 95% CI [0.89‐2.15]) had no significantly higher mortality risk than not fatigued cancer survivors.

**Table 4:** Risk estimates of the association of CRF with all‐cause mortality of cancer survivors not experiencing anhedonia, stratified by cancer type and gender, using imputed data

	Total, N	Deaths, N	Person‐years	Adjusted^a^		
				HR		95% CI
Male colorectal cancer survivors^b^						
Not Fatigued	438	78	2741.8	1.00	—	
Fatigued	165	52	985.6	1.74	1.23‐2.48	
Female colorectal cancer survivors^b^						
Not Fatigued	333	44	2136.30	1.00	—	
Fatigued	182	31	1143.40	1.11	0.70‐1.77	
Endometrial cancer survivors^b^, ^c^						
Not Fatigued	216	34	1719.2	1.00	—	
Fatigued	119	26	927.7	1.38	0.89‐2.15	

FAS total score cut‐offs: not fatigue (10‐21) & fatigue (22‐50).^22^, ^23^

a Analysis was adjusted for age at invitation, cancer stage, primary treatments, years since diagnosis, education, number of comorbidities at invitation, and smoking if appropriate.

b HADS‐cut off: ≥6.^25^

c Smaller sample size as the HADS questionnaire was not part of one study.

## 4 DISCUSSION

This study provides evidence for the association of CRF with all‐cause mortality in male CRC survivors. No association was observed between fatigued survivors and all‐cause mortality in female survivors of CRC or EC. Within EC survivors, only very fatigued EC survivors showed a significant increased risk of all‐cause mortality. The found association in male CRC survivors is in line with the study of Hsu et al,^11^ which showed an association of fatigue with all‐cause mortality (HRadj = 1.76, 95% CI [1.34‐2.95]) in a stage II‐III CRC cohort. However, results in that study were not stratified by gender. Moreover, our result was also similar with results reported for prostate, breast, lung, and esophageal cancer,^7^, ^8^, ^29^, ^30^, ^31^ and for a noncancer population.^32^ However, a study by Maisey et al^10^ found no association of CRF with all‐cause mortality among 501 male and female advanced CRC survivors. The different population (advanced cancer stage, no stratification for gender) may explain the differences in results with our study.

In contrast, although higher CRF prevalence rates were observed among female survivors, we found no significant association of CRF with all‐cause mortality. An exception was among the very fatigued EC survivors where a significant association could be observed. However, the HR should be interpreted with caution as the sample size in this group was very low and the 95% CI was very wide. A possible explanation for this gender difference could be that CRF has a different etiology in women, compared to men.^33^ For example, a study done in a population‐based sample of working age individuals showed that women were more likely to be fatigued than men. Among women, gender‐specific biological complaints and psychological distress were related to fatigue. However, in men, fatigue was related to psychosocial problems, having handicaps and severe chronic complaints.^33^ Cancer survivors with comorbid conditions such as arthritis, hypertension, or cardiac disease were more likely to be fatigued^34^ and have higher mortality risk.^35^ In our study, fatigued males were more likely to have a heart condition ((26.2% vs 18.7%; *P* = 0.011), data not shown).

Another important finding is that the shown association between CRF and all‐cause mortality in male CRC survivors could still be found in the sub‐analysis of nonanhedonic survivors. Among nonanhedonic women, there was no significant association of CRF with all‐cause mortality. However, we did find a significant association of anhedonia with all‐cause mortality in female CRC survivors ((HRadj = 1.60, 95% CI [1.02‐2.50]), data not shown). This association was weaker in male CRC survivors ((HRadj = 1.44, 95% CI [1.05‐1.98]), data not shown). CRF and anhedonia have a similar phenomenology^36^ and tend to co‐occur.^37^ In our previous study,^38^ we identified subtypes of fatigue using the Multidimensional Fatigue Inventory.^39^ Our findings suggest that male and female CRC survivors could experience CRF and symptoms of anhedonia differently as female survivors were more likely to be classified in either the “low fatigue, moderate distress” or “high fatigue, moderate distress” subgroups. Future studies on the association between CRF with mortality could explore whether mortality risks vary with different types of fatigue, for example, physical, mental, or cognitive fatigue.^40^

Our results have clinical implications. First, as we found an association of CRF with all‐cause mortality in male CRC survivors, special attention should be given to that group, particularly when they have a comorbid heart condition. Second, considering CRF is very prevalent and is associated with survivors’ well‐being^41^ and mortality, cancer survivors should be screened for CRF after end of active treatment and survivors suffering from CRF should be advised about fatigue interventions (eg, exercise, pharmacological, psycho‐education, and mind–body therapies).^42^

Several limitations of this study must be considered. CRF was only assessed at one point in time. As a consequence, we cannot take into account potential changes of CRF over time. Similarly, we could not determine whether CRF or heart condition was prior to cancer diagnosis. However, we assessed CRF by time since diagnosis and it was relatively stable (no significant *P*‐values, data not shown), suggesting that our results are robust. Additionally, our study sample is a collection of four separate study samples, with different inclusion criteria and sample sizes. Therefore, we addressed possible survivorship bias by using a left‐truncated Cox hazards regression model. Moreover, information about disease progression was only available for CRC survivors. However, the lack of disease progression data in EC might not be an issue, because these survivors were mainly included within 1 year after diagnosis and mainly with stage I disease, thus having a very low risk of recurrence at the time of survey.^43^

Nevertheless, there are also several strengths of this study. To our knowledge, this is the first study assessing this relationship using a large population‐based study sample, with uniform patient recruitment procedures and the availability to link PRO data with clinical registry data. Additionally, we were able to perform subgroup analysis and specially to stratify our analysis by gender and cancer due to the large sample size, which allowed a more detailed understanding of the association.

In conclusion, results from this large population‐based study contribute to the growing body of knowledge on the association of CRF with all‐cause mortality. We found that CRF is significantly associated with all‐cause mortality in male CRC survivors, irrespective of potential confounders. As CRF is one of the most common distressing symptoms in cancer survivors, health care providers should increase their attention towards the recognition and treatment of this condition. Additionally, we suggest that based on our results further research should assess whether lowering the burden of CRF results in a reduction of all‐cause mortality risk.

## CONFLICT OF INTEREST

Authors have nothing to disclaim.

## DATA AVAILABILITY STATEMENT

The data that support the findings of this study are available on request from the corresponding author. The data are not publicly available due to privacy or ethical restriction.
