# Supplementary material for: The association of cancer‐related fatigue with all‐cause mortality of colorectal and endometrial cancer survivors: Results from the population‐based PROFILES registry
Source: Cancer Med. 2019 Apr 22;8(6):3227–36. doi: 10.1002/cam4.2166 (PMC6558477; doi:10.1002/cam4.2166)
Supplement: Supplementary file 1 [file CAM4-8-3227-s001.docx]

**Appendices**

| **Table S1: Demographic and clinical characteristics of respondents and non-respondents** | | | | |
| --- | --- | --- | --- | --- |
|  | **Total** | **Non-Respondents** | **Respondents** |  |
|  | (n=2,848) | (n=789) | (n=2,059) |  |
|  | **Col%** | **Col%** | **Col%** | **P-Value** |
| **Cancer Survivors** |  |  |  |  |
| Colorectal Cancer | 73.1 | 70.8 | 71.4 |  |
| Endometrial Cancer | 26.9 | 29.2 | 28.6 | 0.22 |
| **Female** | 61.7 | 65.7 | 60.2 | **0.007** |
| **Age at survey** |  |  |  |  |
| ≤ 60 | 28.1 | 21.9 | 30.5 |  |
| 61-70 | 35.3 | 31.3 | 36.8 |  |
| 70+ | 36.6 | 46.8 | 32.7 | **<0.001** |
| Mean (SD) | 66.0 (9.7) | 68.1 (9.9) | 65.2 (9.5) | **<0.001** |
| **Cancer stage** |  |  |  |  |
| I | 45.0 | 40.8 | 46.6 |  |
| II | 32.0 | 34.8 | 31.0 |  |
| III | 22.3 | 22.9 | 22.0 |  |
| Unknown | 0.7 | 1.5 | 0.4 | **0.010** |
| **Years since diagnosis** |  |  |  |  |
| ≤1 | 15.6 | 16.0 | 15.4 |  |
| 2 to 3 | 59.1 | 59.3 | 58.1 |  |
| 4 to 5 | 25.3 | 24.7 | 26.5 | 0.62 |
| **Chemotherapy** | 20.5 | 19.8 | 20.8 | 0.54 |
| **Radiotherapy** | 24.0 | 29.2 | 26.4 | 0.18 |
| Col. - Column | | | | |

| **Table S2: Risk estimates of the association of CRF with all-cause mortality of cancer survivors** | | | | | | | |  |  |
| --- | --- | --- | --- | --- | --- | --- | --- | --- | --- |
|  |  |  |  | **Univariate** | | **Adjusted^1^ (non-imputed)** | | **Adjusted^1^ (imputed)** | |
|  | **Total, N** | **Deaths, N** | **Person-Years** | **HR** | **[95% CI]** | **HR** | **[95% CI]** | **HR** | **[95% CI]** |
| **Colorectal and Endometrial Cancer Survivors** | | | | | | | |  |  |
| Not Fatigued | 1235 | 204 | 7,955.5 | 1.00 | - | 1.00 | - | 1.00 | - |
| Fatigued | 826 | 204 | 5,112.2 | 1.56 | [1.29 – 1.90] | 1.54 | [1.29 – 1.90] | 1.50 | [1.19 – 1.78] |
| Male | 821 | 193 | 4,998.1 | - | - | 1.00 | - | 1.00 | - |
| Female | 1238 | 215 | 8,044.3 | - | - | 0.59 | [0.46 – 0.75] | 0.68 | [0.54 – 0.82] |
| **Colorectal Cancer Survivors** | | | | | | | |  |  |
| Not Fatigued | 886 | 151 | 5,579.8 | 1.00 | - | 1.00 | - | 1.00 | - |
| Fatigued | 575 | 148 | 3,443.4 | 1.60 | [1.28 – 2.01] | 1.60 | [1.27 – 2.03] | 1.57 | [1.24 – 1.98] |
| Male | 821 | 193 | 3,861.4 | - | - | 1.00 | - | 1.00 | - |
| Female | 638 | 106 | 2,641.1 | - | - | 0.58 | [0.46 – 0.75] | 0.60 | [0.47 – 0.77] |
| FAS total score cut-offs: not fatigue (10-21) & fatigue (22-50)(22,23)  ^1^ Analysis was adjusted for age at invitation, cancer type, gender, cancer stage, primary treatments, years since diagnosis, education and number of comorbidities at invitation if appropriate. | | | | | | | | | |
